# Novel Approaches in Ovarian Cancer Research against Heterogeneity, Late Diagnosis, Drug Resistance, and Transcoelomic Metastases

**DOI:** 10.3390/ijms20112649

**Published:** 2019-05-29

**Authors:** Anna Erol, Magdalena Niemira, Adam Jacek Krętowski

**Affiliations:** 1 Clinical Research Centre, Medical University of Białystok, M. Skłodowskiej-Curie 24a, 15-276 Białystok, Poland; magdalena.niemira@umb.edu.pl (M.N.); adamkretowski@wp.pl (A.J.K.); 2 Department of Endocrinology, Diabetology and Internal Medicine, Medical University of Białystok, M. Skłodowskiej-Curie 24a, 15-276 Białystok, Poland

**Keywords:** ovarian cancer, omics, high-grade serous ovarian cancer, genomics, transcriptomics, epigenomics, metastases, cancer stem cells

## Abstract

The development of modern technologies has revolutionised science and has had a huge impact on biomedical studies. This review focuses on possible tools that scientists can use to face the challenges of fighting ovarian cancer. Ovarian cancer is the deadliest gynaecologic malignancy and, even after years of study, the mortality has not decreased significantly. In the era of sequencing and personalised and precision medicine, we are now closer than ever to helping patients and physicians in regard to treatment and diagnosis of this disease. This work summarises the newest findings in the development of ovarian cancer research.

## 1. Introduction

Technology has revolutionised every aspect of our lives. Computers have allowed us to analyse bigger datasets faster. Modern technologies are also strongly present in life sciences. Since Crick and Watson described DNA structure [1] and since it has been recognised as hereditary information [2], extensive studies have been conducted to further understanding on DNA, referred to as “the code of life”. In 1990, the Human Genome Project (HGP) was launched, with the goal of sequencing and mapping the entire human genome. There was much hope and promise in that unveiling of the genome sequence would provide new insights and lead to the development of tools for fighting diseases. In April 2003, the project was successfully finished, earlier and more economically than predicted. However, the results produced many more questions than answers [3].

Molecular mechanisms are multi-layered networks, and many diseases, including cancer, disrupt many of these levels. It is known that the human genome includes coding regions (around 1–2% of the genome) and non-coding regions (98–99%). For a long time, non-coding DNA was considered as “junk” DNA. However, nowadays it is known to have structural and functional relevance [4,5,6]. Sequence variations in coding regions can impact protein structures, and those in non-coding regions may result in differentiated gene expression and splicing. Gene expression describes the dynamic state of cells and tissues. In a diseased state, gene expression is often altered according to different levels of genetic regulation. This can involve regular mechanisms, such as through promoter or enhancer activity, or other mechanisms, such as DNA methylation and the expression of coding and non-coding RNA [7]. After protein synthesis, protein–protein interactions and post-translational modifications (for example glycosylation, phosphorylation, ubiquitination) can strongly impact the correct functioning and structure of proteins [8]. Recently, there has been a rise in multiomics technologies which tend to integrate different levels of so-called “omics”. These omics technologies are collective characterisations and measurements of pools of biological molecules which are then translated into the structure, function, and dynamics of organisms. The basis for omics technologies are next generation sequencing (NGS), mass spectrometry (MS), and antibody-dependent reverse-phased protein microarrays (RPPM).

NGS methods are very useful in cancer biology. Whole-genome and whole-exome sequencing allow for the investigation of somatic mutations related to cancer. Additionally, the use of RNA sequencing allows for the identification of altered gene expression, novel transcripts, and gene fusions. The epigenome plays a crucial role in the regulation of cell function. NGS allows for the study of methylation profiles during tumourigenesis through chromatin immunoprecipitation sequencing (ChIP-Seq), bisulphite sequencing, or methylation-targeting microarray methods. Moreover, NGS can be used for germline genotyping. Germline genotyping is useful for the investigation of cancer predisposition, and can identify not only the germline status of known cancer predispositions, but also identify novel loci of inherited variation. MS-based techniques allow for the identification and quantification of proteins, peptides, and metabolites. Proteins control most of the biological processes and metabolites provide an insight into the state of metabolic pathways and cell fluxes. Changes in the protein or metabolic profiles can give important clues due to understanding the cell state and key disrupted pathways. Additionally, analysing multiomics data in combination with the clinical data from patients will allow us to better understand the risk and protective factors of diseases and improve strategies for their prevention.

This review focuses on new developments and promises of modern technologies for ovarian cancer (OC) research. OC is the most fatal gynaecological malignancy and the fourth leading cause of cancer-related deaths among women in Europe and the United States [9,10]. In 2018, the number of new OC cases worldwide reached almost 300,000, and the number of deaths from OC almost 200,000 [11]. These numbers have not changed much for some years. The high mortality rate of OC is partially attributed to the asymptomatic nature of early-stage OC, which often leads to late diagnosis. Although conventional therapeutic strategies have been developed, the long-term prognosis of OC patients is still poor [12,13,14]. There are no well-established screening methods or specific and sensitive diagnosis tools for the diagnosis of early-stage OC with high accuracy [15,16]. Therefore, novel and effective therapeutic and diagnostic approaches are in urgent demand. Such approaches could be made possible, for example, by identifying new molecular biomarkers or using a combination of biomarkers to obtain fewer false-negative results. Drug resistance often develops during OC treatment, leading to treatment inefficiency [17]. The mechanisms underlying the pathogenesis and reoccurrence of OC are not yet well understood. Understanding the pathways of cancer progression and pathogenesis, and the development of multidrug resistance in OC, will result in new therapies and the discovery of new molecular targets. OC is characterised by high disease heterogeneity, poorly understood progression, and the absence of definite precursor lesions. Different OC subtypes differ in terms of prognosis and response to chemotherapy. Furthermore, the newest studies show large differences in cell origin and epidemiology, as well as the driver mutations of OC histotypes [18,19]. Tumourigenesis can start in epithelial cells, stromal cells, or germ cells [18,19]. However, 85–90% of all OCs are epithelial carcinomas. The four common subtypes of OC are serous, endometrioid, clear-cell, and mucinous cancers [19,20]. The detection of differences in molecular pathways between cancer subtypes will lead to more precise treatment by allowing the correct targets to be accurately targeted. A number of excellent review papers have been published on the pathology and heterogeneity of OC [13,16,19,21,22,23].

## 2. Focus on Genomics

The human genome undergoes sequence alterations, which lead to genetic variations. Such variations can be detected by NGS. One example is point mutations, which involve single-nucleotide base-pair changes (SNPs) and structural rearrangements. Genome variations caused by point mutations can result in benign, protective, or harmful consequences for an organism. Knowledge about any relevant genome changes allows us to better understand the molecular basis of diseases. Recognition of changes in functional genetic variants of a disease can help us simplify diagnosis, guide patient treatment, and recognise genetic risk factors [24]. The integration of genomic profiling with other omics and clinical characteristics allows opportunities for developing precise and personalised treatments, and for the identification of molecular biomarkers for early diagnosis.

During its pan-cancer analysis project, The Cancer Genome Atlas (TCGA) [25] investigated the genomics of high-grade serous ovarian adenocarcinomas (HG-SOC). The investigation included the analysis of expression profiles of messenger RNA (mRNA) and micro RNA(miRNA), the methylation of promoter regions, and DNA copy number variation in 489 HG-SOC samples, and additionally included the analysis of exons in 316 samples. The pathway analysis demonstrated the involvement of notch receptors (notch), forkhead box M1 (FOXM1), RB transcriptional corepressor 1 (RB1), and phosphatidylinositol 3-kinase/RAS type GTPase family (PI3K/RAS) signalling in the pathophysiology of HG-SOC [26]. Data have been published online, and have been used by many scientists for further investigation.

Data analysis is becoming more complex. Studies focus on aberrations in the genome, transcriptome, and epigenome between tumour and control samples. Data of each omics can be analysed individually or in combination, the latter of which is known as multiomics. Additionally, sequencing data can be analysed after integration with clinical data, such as survival outcomes or drug resistance. This helps to understand the key pathways in diseases. The investigation of differences between primary tumour and metastasis sites are bringing researchers closer to understanding tumour evolution. Many scientists are using publicly available data to build new algorithms and data networks for data mining, and are thereby recognising complex patterns and correlations.

### 2.1. Somatic and Germline Mutations, Amplifications, and Structural Instability

An integrated genomic analysis of OC by the TCGA team showed a high prevalence of tumour protein p53 (*TP53*) mutation (96%) in HG-SOC. Also, neurofibromin 1 (*NF1*), BRCA1/2 DNA repair associated (*BRCA1/2*), *RB1*, and cyclin dependent kinase 12 (*CDK12*) were found to have a statistically significant incidence of recurrent somatic mutations, however, their prevalence in the population was much lower [26]. The analysis identified around 113 significant focal DNA copy number aberrations. Furthermore, the survival analysis showed a higher overall rate of survival in the BRCA1/2-mutated (germline and somatic mutations) cases than in BRCA1/2 wild type. In cases of epigenetically silenced BRCA1, which are mutually exclusive to BRCA1/2-mutated cases, the survival outcome was similarly poor as in BRCA1/2 wild type [26]. In around half of the cases, genes which are involved in homologous recombination (EMSY transcriptional repressor, BRCA2 interacting (*EMSY*), phosphatase and tensin homolog (*PTEN*), RAD51 paralog C (*RAD51C*), ATM serine/threonine kinase (*ATM*), ATR serine/threonine kinase (ATR), Fanconi anemia genes) were impaired. This means that the homologous recombination pathway is meaningful, and could be an important target for HG-SOC therapy [26].

Another study used whole-genome sequencing of the tumours of 92 patients with primary refractory, resistant, sensitive, and matched acquired resistant OC to investigate chemoresistance. The results showed that in many cases, chemoresistant tumours inactivated tumour suppressor genes (*RB1, NF1,* RAD51 paralog B *(RAD51B), PTEN*) through gene breakage. Furthermore, they showed that cyclin E1 (*CCNE1*) amplification occurred in samples of refractory disease and primary resistant cells. Additionally, in some individual cases, multiple independent reversions of germline *BRCA1* or *BRCA2* mutations led to promoter fusion, influencing the expression of drug efflux pump *MDR1* [27].

Many patterns can be revealed using modern technologies. Computers allow complicated patterns to be uncovered in large amounts of data. For instance, Macintyre et al. [28] studied the copy number signatures of HG-SOC using shallow whole-genome sequencing of 117 samples. Profiles of copy number signatures at the point of OC diagnosis may correlate with overall survival and the probability of platinum-resistant relapse. OC is characterised by high chromosomal instability [29]. Specific copy number features may allow the prediction of structural changes, such as breakage–fusion–bridge cycles or tandem duplications. For this reason, the following characteristics of copy number features were included into the signatures: the breakpoint number per 10 Mbp, the copy number of the segments, the difference in copy number between adjacent segments, the breakpoint counts per chromosome arm, the lengths of oscillating copy number segment chains, and the size of segments [28]. *TP53* mutation has been shown to be an early event in HG-SOC. However, after analysing HG-SOC signatures, Macintyre et al. [28] described seven different copy number signatures. This suggests that after the initiating *TP53* mutation, multiple different mutational processes follow [28]. The results of copy number signature analysis confirmed the earlier described results which showed that mutations of *BRCA1/2* are related to better prognosis in HG-SOC. A high exposure to signatures characterised by *BRCA1/2*-related homologous recombination deficiency was associated with longer overall survival. Patients with high exposure to signatures with mutations of the RAS pathway (*NF1*, KRAS proto-oncogene, GTPase (*KRAS*), NRAS proto-oncogene, GTPase (*NRAS*)) showed fast platinum-resistant relapse and a poor overall outcome [28]. This information allowed further research to be focused on aberrations in the OC genome which influence patient survival or therapy response success. Knowledge about the main pathways involved in OC pathogenesis will assist in the development of suitable and precise therapeutics.

### 2.2. Tumour Evolution and Heterogeneity

OC varies according to histological type and grade in genetic characteristics, precursor lesions, response to treatment, and patient outcome [30]. The most common OCs are epithelial ovarian cancers (EOCs). Around 50–60% of EOC cases are HG-SOC, and these are the best studied among all OCs. The integrated genomic analysis of HG-SOC by TCGA is currently the biggest genomic analysis for OC. Many of the molecular differences that exist between the histotypes of EOC have been characterised in genomics studies. Table 1 summarises the most common mutations and pathway alterations for different types of EOC. HG-SOC is associated with chromosome structure instability and initiating *TP53* mutations. Furthermore, homologous recombination repair genes are also altered in many cases of HG-SOC [26,28]. HG-SOC is the most aggressive subtype of EOC, and is often discovered when already at an advanced stage. Around 5% of EOCs are low-grade serous ovarian cancers (LG-SOC). Genetic profiling of LG-SOC has shown less overall karyotype instability and lower rates of mutation than in HG-SOC. Mutations in B-Raf proto-oncogene, serine/threonine kinase (*BRAF*), and KRAS are the most abundant mutations in the LG-SOC subtype. Abnormalities in these genes cause the constitutive activation of the mitogen-activated protein kinases/extracellular signal-regulated kinase (MAPK/Erk) pathway. The activation of downstream processes of this pathway results in higher tumour cell survival and proliferation. Another type of EOC is endometrioid carcinoma. This subtype accounts for around 25% of EOC cases. Characteristics of endometrioid carcinomas are an impaired PI3K pathway and an aberration of the main effector of WNT signalling gene family/catenin beta 1 (WNT/CTNNB1) signalling [31,32]. Studies on mucinous epithelial carcinoma have often shown mutations within the RAS signalling pathway [33]. The last EOC subtype to be introduced is clear-cell carcinoma, which often acquires inactivating mutations in the switch/sucrose non-fermentable chromatin remodelling complex (SWI/SNF) [13,34].

Only a few studies have compared the overall genetic and expression profiles between primary tumours and metastases. Lee et al. [45] investigated genomic changes during tumour evolution. Whole-exome sequencing with a high depth of coverage was used to investigate the variants of mutations in metastatic sites [45]. Next, targeted ultra-deep sequencing was conducted to study somatic mutations and perform copy number analysis. Study material was collected from the primary tumour sites and associated metastatic sites of a HG-SOC patient (Staging of The International Federation of Gynecology and Obstetrics (FIGO) IIIC). To investigate the evolution of the tumour, a phylogenetic tree was generated. The results of the analysis were consistent with those of previous studies in that mutation of TP53 was ubiquitous in all metastatic and primary tumour samples. The phylogenetic analysis based on mutation profiles identified two clusters of primary tumours (P1 and P2) and one of the metastatic (M) regions. The two clusters of primary tumours had already diversified in the early phase of tumourigenesis. The M cluster originated in the P1 primary tumour cluster, and only a few additional somatic mutations and copy number variations arose due to metastatic processes [45]. The authors suggested that in the examined patient, transcoelomic metastasis arose with little accumulation of somatic mutations and copy number alterations. The patient did not show tumour reoccurrence for at least 12 months after cytoreduction surgery [45]. A follow-up and comparisons with a bigger cohort could allow for a good overview of tumour evolution in relation to outcome. Another group performed phylogenetic analysis of HG-SOC using multiregion whole-genome and single-nucleus sequencing of 68 samples from seven patients to reveal the characteristics of cell populations spread within HG-SOC. The results supported the findings of Lee et al. [45]. A high degree of polyphyletic clonal mixing and reseeding of clones at distal foci was not often observed. Mostly, clonal diversity had already emerged at a primary site, and the seeding followed in a unidirectional, monoclonal way to distal intraperitoneal sites [46]. High intratumoural heterogeneity of OC has been observed in the personal landscapes of somatic mutations, copy number alterations, transcriptome variations, and gene aberrations in primary and metastatic sites [47].

## 3. Focus on Transcriptomics

Cell mechanisms occur on many levels, and are strictly regulated to ensure the proper functioning of cells. Transcriptomics examines RNA expression levels. This includes both mRNA and non-coding RNAs (ncRNA). ncRNAs are involved in regulatory events. Complex statistical data analysis allows for the classification of diseases and prediction of their future outcome, in addition to facilitating a better understanding of the underlying biological disease mechanisms [24].

Zhu et al. [48] presented a complex analysis using multiomics with clinical data for various human cancers for quantifying the prognostic values of omics profiles via kernel machine learning. The best performance regarding the accurate prediction of clinical variables (e.g., survival) was seen when using mRNA, miRNA, and methylation profiling. However, the prediction performance increased even more when multiomics was included as part of the kernel machine learning method [48].

An analysis of differentially expressed genes in OC and their relation to patient survival was performed by Hossain et al. [49] using data published in the TCGA database. The analysis focused on profiling 26 genes which had previously been mentioned in the literature as being important in OC biology. Using multivariate, univariate, and combined analysis, they identified genes which may be critical markers for the progression and survival of OC. Kallikrein-6 (KLK6), which is a member of the kallikrein-related peptidase family, showed significantly different expression levels in all three analyses [49]. This result was consistent with previous studies which showed the involvement of KLK6 in OC aggressiveness [50,51]. Modern data science approaches allow for the mining of existing data. The databases of TCGA and the Gene Expression Omnibus (GEO) have been used to build integrated competing endogenous RNA (ceRNA) networks for OC with the goal of predicting candidate RNA signatures for recurrent disease [52]. The treatment of OC faces many challenges. One of them is drug resistance. Knowledge regarding the development of drug resistance in OC is limited. Some of the factors influencing the reoccurrence and drug resistance of OC are altered miRNA and long non-coding RNA (lncRNA) expression levels [17,53]. miRNAs are short, single-stranded non-coding RNA molecules that play an important role in the post-transcriptional and transcriptional expression of genes. lncRNAs are long (>200 nt) non-coding transcripts that play a role in OC pathogenesis, especially in epithelial–mesenchymal transformation (EMT) [17,53]. Wang et al. [52] used a support vector machine classifier to analyse the expression patterns of mRNA, miRNA, and lncRNA in OC patients with the goal of identifying prognostic RNAs for disease reoccurrence and to build a network to reveal the potential regulatory relationships between various RNAs. A significant difference was found in the expression of 36 genes (e.g., *TP53*, RNA binding protein, mRNA processing factor (*RBPMS*)). The results showed differential expression of three lncRNAs and many miRNAs. Significant alterations in the expression of these genes could be potential biomarker for OC reoccurrence. The ceRNA network revealed a connection with the altered expression of *RBPMS* and *TP53* genes. These genes and their interaction with miRNAs may be a crucial mechanism underlying OC reoccurrence, and should be further investigated [52].

Many studies show the involvement of miRNAs in OC pathogenesis. For example, miR-542-3p has been shown to be strongly downregulated in EOC. Functional analysis of the miRNA showed that overexpression of miR-542-3p results in the suppression of tumour progression. Additionally, knockdown of that miRNA supports a role tumour development. One of the targets of miR-542-3p is cyclin-dependent kinase 14 (*CDK14*). *CDK14* is directly involved in the control of the eukaryotic cell cycle [54] and, therefore, any abnormalities in its regulation may negatively impact the cell cycle.

miRNAs are also strongly involved in the coordinating network of DNA damage response (DDR). An miRNA expression-dependent model, based on 10-miRNA-score, has been developed for the prediction of OC outcomes [55]. The building of an RNA network includes many steps, starting with obtaining genes involved in DDR. The miRNAs that interact with the obtained genes have been searched for and carefully chosen using strict rules. Next, the miRNA regulatory network was built. This included miRNA—target interactions between 75 miRNAs and 55 DDR genes. The index of genome instability was evaluated using the correlation between altered miRNA expression and the frequency of DDR gene mutation. The 10-miRNA-score model was constructed using 10 miRNAs which showed a significant and strong correlation regarding their level of DDR gene mutation in cancerous genomes. This allows for the prediction of defects in DDR and genome instability. The verification of the model showed that a low 10-miRNA-score predicts poor survival of the patients [55]. Elias et al. [56] also focused on using miRNAs as prediction markers, and developed an miRNA algorithm for the diagnosis of EOC. The algorithm—developed using machine learning—is able to discriminate between benign, non-invasive tumours, and healthy samples. The resulting neural network consists of 14 miRNAs and seven neurons in the hidden layer. The data used for the analysis came from three independent databases (Effects of Regional Analgesia on Serum miRNA after Oncology Surgery Study (ERASMOS), Pelvic Mass Protocol (PMP), and New England Case Control Study (NECC)) and included 179 patients. The algorithm was tested during one clinical study. The results outperformed currently used diagnostic tools, suggesting potential for this approach to be used in non-invasive diagnostic testing for OC [56]. Many other attempts of using miRNA networks have been described and show great potential as predictive and prognostic biomarker [57,58].

In order to develop effective treatment, it is important to target key mutations underlying disease development that are also amenable to drug treatment. Strong inter- and intratumoural heterogeneity may lead to the different, and sometimes completely opposite, behaviour of specific alterations in one subtype of cancer compared to another. For this reason, drugs that help in one cancer might not be useful in treating another. This also explains the importance of complex multifactorial analysis [34,59]. To reveal OC intratumoural heterogeneity, Shih et al. [60] investigated tumour cell populations using single-cell RNA sequencing (scRNA-seq). They studied cell population patterns and their changes between different grades of disease (low, high, benign) as well as in primary versus metastatic sites. The study included 14 samples from nine patients. A total of 16 different cell clusters were identified, and specific cells were found to be correlated with differences in tumour grade. Moreover, the authors identified changes in the proportion of epithelial cells to leukocytes from primary and metastatic sites. Additionally, in samples of primary tumour, myeloid lineage cells were shown to be the main cell population which expresses soluble factors, while in metastatic samples, these factors were mainly expressed by fibroblasts. Furthermore, it was found that leukocytes were not suppressed by pro-tumour cytokines in any of the cell populations.

The analysis was based on four subtypes within HG-SOC (differentiated, immunoreactive, mesenchymal, and proliferative) as described by Bell et al. [26]. The differentiated subtype cell population was most common in epithelial cells of benign tumours and LG-SOC. The immunoreactive subtype was most abundant in the myeloid lineage cells of primary and metastatic tumours. The mesenchymal subtype characteristics were connected to primary fibroblasts, metastatic fibroblasts, and cancer stromal cells. No enrichment of the proliferative subtype was observed [60].

As mentioned before, OC tumours are very heterogeneous, containing many subpopulations. Cancer stem cell populations with stem cell-like characteristics have been extensively studied in recent years, and may play a very important role in cancer progression, drug resistance, and the metastasis process [61,62]. The scRNA-seq technique allows tumours to be examined at single-cell resolution, which allows for new insights into tumour biology [60].

Tumour progression models based on cancer stem cells are of increasing interest. Cancer stem cells are self-renewing, apoptosis-resistant, able to differentiate asymmetrically, often resistant to chemotherapy and radiotherapy, able to form spheroids, and have tumour-initiation ability. Cancer stem cells are thought to persist in tumours as small populations, and are considered to cause relapse and metastasis. OC is described as a prototypical example of a cancer stem cell-driven disease. Cells found in peritoneal ascites are cancer cells with the ability to overcome anoikis, as has been shown in vitro. In vivo, the cells show the ability to develop the tumour and metastasise [62,63].

Understanding the biology of OC stem cell populations and its role in disease progression may be crucial to resolving most difficulties in the treatment of ovarian cancer, such as drug resistance, cancer relapse, and metastasis. The omics approach, together with appropriate downstream analyses, may be helpful in identifying cancer stem cells. The scRNA-seq technique allows cell population heterogeneity to be elucidated [60], which can help in analysing cancer stem cell populations and their fluctuations during different stages of OC. As computational methods to study cancer cell hierarchies are developing quickly and are becoming more advanced, a method has been introduced to extract cell type-specific gene expression signals from the gene expression profiles of unsorted tumour cells by deconvolution. One group applied this method to study acute myeloid leukaemia and recover markers for acute myeloid leukaemia stem cells [64]. Using bulk sequencing results, they were able to provide specific gene expression profiles for cancer stem cells and propose potential markers for them.

## 4. Focus on Epigenomics

DNA expression is regulated very tightly and on many levels. Alterations in the regulation processes lead to abnormalities in cell function. The epigenome regulates gene expression without altering the primary DNA sequence. An important component of the epigenome is miRNA, which plays a role in post-transcriptional regulation. Epigenetic modification also includes DNA methylation and histone modification. These processes modulate gene expression at the transcription level. DNA methyltransferase is an enzyme which adds methyl groups to cytosine nucleotides, commonly on cytosine–guanine dinucleotides (CpGs). The unique level and pattern of DNA methylation plays an important role in tissue-specific expression, suppression of the expression of repetitive elements, allele-specific expression, as well as the inactivation of the X-chromosome in women. Changes in the epigenetic state may cause alterations to fundamental cell functions and trigger diseases such as cancer. Commonly observed methylation patterns in cancer are (1) hypermethylation in the promoter regions of tumour-suppressing genes, and (2) the hypomethylation of highly repetitive DNA sequences [17,65,66,67,68].

Earp et al. [30] reviewed alterations in the hypermethylation of DNA between EOC histotypes. They noticed strong variations in the results for histotype-specific DNA methylation. Since DNA methylation is tissue-specific, correct matching of precursor cancer tissues to control tissues is crucial for obtaining correct results. However, the identity of the precursor tissue is not always clear, which imposes a strong limitation to epigenomic studies of OC. Additionally, it is known that the patterns of DNA methylation become less consistent with increasing age, and that OC is more common in postmenopausal women. The small sample size of rare OC histotypes and the lack of replication in the associated studies are other issues leading to the high variation in methylation profiles between the studies. It is necessary to find a way to overcome these limitations in order to draw conclusions regarding the patterns of DNA methylation between OC histotypes [30]. However, histological type does not seem to influence the global DNA hypomethylation of repeat elements, which has been proposed to be a ubiquitous characteristic of cancer [30].

Sina et al. [65] introduced a simple and quick assay for detecting cancer based on the differences in physicochemical properties between normal and epigenetically altered cancer genomes. Cancer DNA is globally hypomethylated, with short regions of clustered methylation often occurring in regulatory regions. These characteristics lead to purified cancer genomic DNA (gDNA) dissolving in solution, and also to higher DNA–gold affinity, properties which were exploited for the detection of cancer, however, further analysis is needed to describe the type, stage, and likelihood off disease recurrence is. Table 2 summarises the characteristics of cancer and normal genomes that are important for this cancer diagnosis method [65].

Unlike changes in DNA sequences, DNA methylation and histone modification are reversible. Therefore, the investigation of epigenetic alterations in OC progression could lead to novel treatments being developed. Epigenetic therapies target DNA methyltransferase (DNMT) and histone deacetylase (HDAC). The DNMT inhibitor (DNMTi) decitabine was investigated in a clinical trial of 17 heavily pretreated and platinum-resistant patients. The study showed that decitabine helped to restore sensitivity to carboplatin and resulted in a high response rate (35%) and prolonged progression-free survival (10.2 months). The patients with restored sensitivity had a greater number of demethylated genes, including the known tumour suppressor genes mutL homolog 1 (*MLH1*), Ras association domain family 1 isoform A (*RASSF1A*), and homeobox A10 and -A11 (*HOXA10* and *HOXA11*) [69]. However, most of the clinical trials using single-agent therapy had disappointing outcomes. A new approach is combination therapy, which uses several epigenetic drugs. The combination of the pan-HDAC inhibitor belinostat with DNMTi decitabine was shown to enhance the effects of single-agent therapy both in vitro and in mice [70,71]. A targeted treatment approach involving the inhibition of HDAC6 in tumours with mutated AT-rich interaction domain 1A (ARID1A) showed an improvement of survival using xenograph. This success was due to the direct deacetylation of Lys120 of cellular tumour antigen p53 (p53), resulting in selectively promoted apoptosis [72].

A better understanding of the molecular mechanisms and patterns of the epigenome will allow for more effective usage of existing therapies and for the development of novel therapies dependent on cancer characteristics.

## 5. Focus on Proteomics and Metabolomics

Useful complementation and validation to genomics and transcriptomics information are proteomics and metabolomics information. The analysis of the genome, gene expression, and its regulation create many hypotheses about cell processes. Given that proteins control most of the biological processes, proteome alteration can give additional insight into pathways which drive diseases, whereas endogenous metabolites influence biochemistry of cell processes. Proteomics and metabolomics methods allow the identification and quantification of small molecular compounds, description of protein–protein interactions, identification of posttranslational modifications, and functional analysis, and are needed for the translational success of cancer genomics [8]. Nevertheless, there are still many technical difficulties to overcome. Proteins are very dynamic and there are no tools to amplify them yet. Modern MS approaches allow accurate analysis of differential protein abundance, genome-wide differential protein expression analysis and differential metabolite expression analysis. Notably, the introduction of high-resolution “Orbitrap” MS instrument working together with computational platforms (f.eg. MaxQuant) allowed for the construction of the first overview of the human proteome [73,74]. Although there have been strong improvements, methods are still under development and constant refinement, and are presently unready for being used routinely in clinical settings and are insufficient for detection of less abundant proteins and metabolites. Less abundant proteins and metabolites are crucial as potential biomarkers, as OC is characterised by loss-of-function and downregulation of tumour suppressor activities [26]. On the other hand, the technologies give promise to a better understanding of cancer processes and pathways, especially when considered together with other omics. This knowledge may lead to the identification of novel, specific and sensible diagnostic and prognostic biomarkers [75]. Given the central dogma model of molecular biology [76], proteins are functional mediators in phenotype characterisation. That means, although decisions are initiated already at the genomic and transcriptional levels, they are executed on the protein level and alteration in the protein level will lead to improper or altered functioning of cell machinery [75,77]. Both proteins and metabolites profiles are altered in cancer cells in comparison to healthy cells and the information gives important insights into the pathophysiology of cancer.

In order to complement the TCGA analysis of HG-SOC genome, the Clinical Proteomic Tumor Analysis Consortium (CPTAC) performed a MS-based proteomic and phosphoproteomic study on samples of HG-SOC. The results exhibit 9600 proteins from 174 tumours and 24,429 phosphosites from 6769 phosphoproteins in a subset of 69 tumours. The integration of proteome information with previously studied genomic information lead to the conclusion, that copy-number alterations influence differences in the proteome, which alters signaling pathways. Homologous repair deficiency, which is widely present in HG-SOC, correlates with acetylation of histone H4. Additionally differences in protein and phosphoprotein abundance indicate the signaling changes, which correlate with survival [78].

Garg et al. [79] conducted metabolomic profiling of HG-SOC and LG-SOC, combining two methods: 1D H Nuclear magnetic resonance spectroscopy (NMR) and targeted MS. The results showed 22 metabolites, which were overlapping in both platforms, and most of the identified metabolites were identified only on one of the platforms. Nowadays, no single platform is able to detect all metabolites in a biological sample, especially due to the high variety of physiochemical properties of metabolites. To achieve more comprehensive profiling, the group used two platforms. Due to this experimental design, they were able to find differences in ascorbate and aldarate metabolism which have not been previously described in EOC. [79]. Additionally, glycophospholipid, serine, cysteine, taurine, tryptophane, fatty acid, choline phospholipid, nitrogen and methane metabolism pathways and glycerol, phosphocholine, ketone bodies concentrations between HG-SOC, LG-SOC and controls differed. The experiment was performed on a small number of patients (*n* < 20 in each group), which is a strong limitation. Further research is necessary to validate the findings and provide its clinical relevance.

Ovarian cancer is characterised by an intertumoral and intratumoral heterogeneity. Experiments conducted on cell lines need to be carefully thought through and appropriate cell lines need to be selected. Experimental design is crucial, otherwise there can be rough consequences for patients if the wrong conclusions have been drawn. Coscia et al. [80] used single-run MS to analyse proteome profiles of 26 OC cell lines, HG-SOC tumours, immortalised ovarian surface epithelial cells, and fallopian tube epithelial cells. Deep proteomics results allowed grouping of the cell lines in three distinct categories: epithelial, clear cell, and mesenchymal cells lines. Additionally, a signature based on 67 proteins has been developed, which is clearly separated for HG-SOC on epithelial and mesenchymal clusters. The two groups vary in survival as well. These results may be another clue confirming the dualistic precursor model of HG-SOC [80].

Combined proteomics and metabolomics studies can be used for the validation of one another and also for better elucidation of mechanisms driving ovarian cancer. Drug resistance development is very common in ovarian cancer and is one of main issues to overcome in the quest for successful treatment. Studies combining proteomics and metabolomics of serum from platinum-resistant and platinum-sensitive EOC have been conducted in order to detect the pathways altered in chemoresistancy and develop detection markers or treatment targets for drug resistant ovarian cancer. A total of 248 proteins have been identified, from which the altered expression of fibronectin 1(FN1), serpin family A member 1 (SERPINA1) and orosomucoid 1 (ORM1) in further analysis showed significant differences in abundance between platinum-resistant and -sensitive samples. However only the ORM1 showed potential to become a sensitive and specific for platinum resistance biomarker with the Area Under The Curve (AUC) for *ORM1*, 0.91. Using High performance liquid chromatography- mass spectrometry (HPLC-MS), 25,800 metabolic features have been revealed and six of them were chosen as candidate biomarkers to develop metabolic signature. Using NMR, metabolic signatures have been built and a clear separation of control group, platinum-sensitive group, and platinum-resistant group has been shown. Metabolites from signature are potential proteomics and metabolomics serum biomarkers for recognition of chemotherapy resistant cancer, which could help future treatment decision-making [81]. The topic of cancer stem cells is also strongly researched in metabolomics. One theory proposes that early and late metabolomic hits can affect chromatin organisation and activate an epigenetic program involved in metabolic-driven reprogramming of cancer stem cells. In this case, identification of key metabolic pathways would be crucial for the identification and targeting of cancer stem cells. Cancer stem cells, sometimes also called tumour initiating cells, are a small but heterogeneous cell population which show stem cell-like characteristics. Additionally, they are thought to be responsible for drug resistance and metastasis. Within the tumour microenvironment, a growing tumour faces hypoxia or limited nutrient availability, and to survive and further grow it need to overcome these problems. Metabolic heterogeneity and plasticity might play a crucial role in tumour adaptability. De Francesco, E.M. et al. [82] pointed out the promising benefits of cancer stem cells metabolomics research. Cancer stem cells, when activated, lead to cancer relapse and in many cases to drug resistance. Understanding cancer stem cell biology and identifying novel treatment targets may strongly influence the success in the handling of cancer patients. It has been asserted that mitochondrial biogenesis and activity plays a critical role in the transition from a quiescent to active state of cancer stem cells [83]. According to the metabostemness theory, there are two main pathways in cancer stem cell metabolism—glycolysis and oxidative phosphorylation—which are important in therapeutic intervention [82,84]. Understanding the metabolic changes of cancer stem cells might help us track and develop targeted therapies to inhibit cancer relapse. The presented study examples demonstrate the potential of the metabolomic and proteomic approaches. The fast technological development of metabolomic and proteomic applications gives hope for future OC research.

## 6. Conclusions

Studies using modern technologies generate large amounts of data, however, the next step—making sense of the data and using this to develop applications—is crucial. A well-prepared experimental design is crucial to facilitate a better understanding of heterogeneous OC. The treatment of OC faces many problems. Inter- and intratumoural heterogeneity results in difficulties for targeted treatment. Asymptomatic early-stage OC often results in late diagnosis. The five-year survival rate of late diagnosed patients is 30%. Most patients develop resistance to chemotherapy after some rounds. Understanding the mechanisms behind this resistance development will allow us to overcome the associated problems of its treatment and target the disease using precise treatment strategies. During tumour progression, somatic mutations, gene expression alterations, epigenetic changes, and chromosomal abnormalities occur, which could be tracked using NGS technologies and subsequent data analysis. It is often hard to clearly identify precursor lesions, which makes the study of tumour evolution complicated [61]. Table 3 summarises the main problems which researchers need to resolve when studying OC, and examples of methods to understand the roots of these problems. In this review, we have discussed modern biomedical approaches which allow us to face main OC challenges.

## Figures and Tables

**Table 1:** The main subtype-specific mutations and other alterations of epithelial ovarian cancer (EOC).

Subtype of EOC	Mutations	Other	Publications
HG-SOC	*TP53, BRCA1/2, NF1, CDK12, RB1, PTEN, RAD51B*, homologous recombination repair genes	PI3K/Ras, notch, FOXM1 pathways alterations	[13,26,27,35,36,37]
LG-SOC	*BRAF, KRAS, NRAS, ERBB2*		[13,35,36,38,39]
Endometrioid	*ARID1A, PIK3CA, PTEN, PPP2R1A*, β-Catenin	MMR deficiency, microsatellite instability	[13,31,35,36,40]
Clear cell	*PIK3CA, PTEN, CTNNB1, PP2R1A ARID1A, TP53,* SWI/SNW	Chromatin remodelling factor inactivation, microsatellite instability	[13,31,35,36,41,42,43]
Mucinous	*KRAS, ERBB2*		[13,33,35,36,43,44]

HG-SOC: high-grade serous ovarian cancer; *TP53*: tumour protein 53; *BRCA1/2*: BRCA1/2 DNA repair associated; *NF1*: neurofibromin 1, *CDK12*: cyclin dependent kinase; *RB1*: RB transcriptional corepressor 1, *PTEN*: phosphatase and tensin homolog; *RAD51B*: RAD51 paralog B PI3K/Ras: phosphatidylinositol 3-kinase/RAS type GTPase family; notch: notch receptors; FOXM1: forkhead box M1; *BRAF*: B-Raf proto-oncogene, serine/threonine kinase; *KRAS*: KRAS proto-oncogene, GTPase; *NRAS*: NRAS proto-oncogene, GTPase; *ERBB2*: erb-b2 receptor tyrosine kinase 2; *ARID1A*: AT-rich interaction domain 1A; *PIK3CA*: phosphatidylinositol-4,5-bisphosphate 3-kinase catalytic subunit alpha; *PPP2R1A*: protein phosphatase 2 scaffold subunit Alpha; *CTNNB1*: catenin beta 1; SWI/SNW: switch/sucrose non-fermentable chromatin remodelling complex; MMR: mismatch repair; LG-SOC: low-grade serous ovarian cancer.

**Table 2:** The characteristics of cancer genomes vs. normal genomes important for the DNA–gold affinity for cancer diagnosis. gDNA: genomic DNA; CpGs: cytosine–guanine dinucleotides [65].

Genome Type	Methylation of Intergenomic Regions	Methylation of Regulatory Regions	Methylscape Biomarker	In-Solution Properties of Purified gDNA	Surface-Based Properties
**Cancer genome**	Low methylation	High methylation	Clustered methylation	DNA solvation	High adsorption
**Normal genome**	High methylation (individual CpGs ~150 kbp apart)	Low methylation	Dispersed methylation	DNA aggregation	Low adsorption

**Table 3:** Overview of the different approaches to resolving problems in the analysis and treatment of ovarian cancer using modern technologies included in this paper. MS—mass spectrometry; miRNA—micro RNA, gDNA—genomic DNA, mRNA—messenger RNA, lncRNA—long non-coding RNA; LC-ESI-MS/MS—liquid chromatography-electrospray ionization/multi-stage mass spectrometry; LC-MS—liquid chromatography-mass spectrometry; RT-PCR—real-time PCR; DNMT—DNA methyltransferase; SNV—single nucleotide variation; CNV—copy number variation;

Problems	Approach	Method	Expected Application	Example Studies
**Heterogeneity**	Studying cell population patterns between ovarian cancer tumours of different grade, as well as between primary and metastatic tumours	Single-cell RNA sequencing	Understanding the leading cell population; may conclude in finding a specific target for diagnosis and precise treatment	[60]
	Proteomic profiling and statistical comparison between ovarian cancer cells and controls	Single-run MS	Potential biomarkers for diagnosis or outcome prediction	[80]
**Late diagnosis**	Training of machine to become a neural network with the lowest number of miRNAs needed for best diagnosis by correlation with clinical data	Machine learning algorithm based on miRNA expression data (microarrays, RNA sequencing)	Building of sensitive non-invasive diagnostic tools	[56]
	Using the physicochemical properties between alterations in genome methylation and gold surface	gDNA isolation and DNA–gold affinity	Development of easy, fast, and non-invasive diagnostic tools	[65]
**Drug resistance**	Building of endogenous RNA network	Support vector machine classifier (using data of mRNA, miRNA, and lncRNA vs. clinical data)	Development of a good model to predict disease reoccurrence in advance and to find potential biomarkers for the development of drug resistance	[52]
	Proteomic and metabolomics investigation and further statistical analysis to recognise differences between controls, platinum-resistant tumour, and platinum-sensitive tumour	2D-LC-ESI-MS/MS, LC-MS	Development of biomarkers for recognition of chemoresistant ovarian cancer	[81]
	Comparison of the primary sensitive and refractory resistant tumour	Whole-genome sequencing; transcriptome, methylation, and microRNA (miRNA) expression analyses	Designing of novel drugs for resensitisation or targeted therapy	[27]
**Metastasis**	Phylogenetic analyses identifying constituent clones and quantifying their relative abundances at multiple intraperitoneal sites	Whole-genome and single-nucleus sequencing	Understanding the process of metastasis migration and understanding the population spread, which could lead to better treatment management in the future	[46]
	Comparison of the mutation landscape, and copy number analysis between primary and metastatic sites	High-depth whole-exome sequencing	Understanding the ways of genomic evolution in transcoelomic metastasis	[45]
	Establishment, isolation, cloning, and propagation of the cellular content of ovarian multilayered spheroids (cancer stem cells) to study their clonogenic, tumourigenic, and invasive properties	In vitro and in vivo study, RT-PCR	Describing cellular mechanisms and the influence of cancer stem cells on the aggressiveness of ovarian cancer	[63]
**Targeting**	Treatment of heavily pretreated and chemoresistant patients with the addition of DNMT inhibitor	Clinical trial	Development of treatment which helps to restore the sensitivity to carboplatin (classic treatment)	[70,71]
	Finding SNV, CNV, alteration in mRNA expression, miRNA expression	Exome sequencing, RNA sequencing, integrated data analysis	Finding driver mutations and key disrupted pathways in pathogenesis for precision medicine	[26,49]
	Analysis of copy number signatures (including many copy number features)	Shallow whole-genome sequencing	Finding ways to predict overall survival and the probability of drug-resistance and relapse at the point of diagnosis	[28]
	10-mRNA-score model constructed so that it strongly correlates with the level of DNA mutations and predicts the genome instability	Construction of RNA network	Prediction model of poor outcome, which could identify important pathways for targeting disease	[55]

## Abbreviations

ARID1A	AT-rich interaction domain 1A
ATM	ATM serine/threonine kinase
ATR	ATR serine/threonine kinase
AUC	Area Under The Curve
BRAF	B-Raf proto-oncogene, serine/threonine kinase
BRCA1/2	BRCA1/2 DNA repair associated
CA125	cancer antigen 125
CCNE1	cyclin E1
CD133	prominin 1
CD44	CD44 molecule (Indian blood group)
CDK12	cyclin-dependent kinase 12
CDK14	cyclin-dependent kinase 14
ChIP-Seq	chromatin immunoprecipitation-sequencing
CNV	copy number variations
CpG	cytosine–guanine dinucleotides
CTCs	cancer tumour cells
ctDNA	circulating tumour cells
CTNNB1	catenin beta 1
DDR	DNA damage response
DNMT	DNA methyltransferase
DNMTi	DNA methyltransferase inhibitor
EMSY	EMSY transcriptional repressor, BRCA2 interacting
EMT	epithelial–mesenchymal transition
EOC	epithelial ovarian cancer
EpCAM	epithelial cell adhesion molecule
ERASMOS	Effects of Regional Analgesia on Serum miRNA after Oncology Surgery Study
ERBB2	erb-b2 receptor tyrosine kinase 2
FIGO	The International Federation of Gynecology and Obstetrics
FN1	fibronectin 1
FOXM1	forkhead box M1
gDNA	genomic DNA
GEO	Gene Expression Omnibus (database)
HDAC	histone deacetylase
HGP	The Human Genome Project
HG-SOC	high-grade serous ovarian cancer
HOXA10/11	homeobox A10/A11
HPLC	High-performance liquid chromatography
IL6/JAK/STAT3	interleukin 6/Janus kinase/signal transducer and activator of transcription 3
KLK6	kallikrein-6
KRAS	KRAS proto-oncogene, GTPase
LG-SOC	low-grade serous ovarian cancer
lncRNA	long non-coding RNA
M	metastatic region
MAPK/Erk	mitogen-activated protein kinases/extracellular signal-regulated kinase
MDR1	multidrug resistance protein 1
miRNA	microRNA
MLH1	mutL homolog 1
mRNA	messenger RNA
MS	Mass Spectrometry
ncRNA	non-coding RNA
NECC	New England Case Control study
NF1	neurofibromin 1
NFκB	Nuclear Factor Kappa B
NGS	Next Generation Sequencing
NMR	Nuclear magnetic resonance spectroscopy
NRAS	NRAS proto-oncogene, GTPase
OC	ovarian cancer
ORM1	Orosomucoid 1
P1/2	primary tumour site
PI3K/AKT	phosphatidylinositol 3-kinase/protein kinase B
PI3K/RAS	phosphatidylinositol 3-kinase/RAS type GTPase family
PIK3CA	phosphatidylinositol-4,5-bisphosphate 3-kinase catalytic subunit alpha
PMP	pelvic mass protocol
PPP2R1A	protein phosphatase 2 scaffold subunit Alpha
PTEN	phosphatase and tensin homolog
RAD51B	RAD51 paralog B
RAD51C	RAD51 paralog C
RASSF1A	Ras association domain family 1 isoform A
RB1	RB transcriptional corepressor 1
RBPMS	RNA-binding protein, mRNA processing factor
scRNA-Seq	single-cell RNA sequencing
SERPINA1	serpin family A member 1
SNPs	single-nucleotide polymorphisms
SNV	single nucleotide variant
SWI/SNF	switch/sucrose non-fermentable chromatin remodelling complex
TCGA	The Cancer Genome Atlas
TP53	tumour protein 53
WNT	WNT signalling gene family
Wnt/β-Catenin	canonical Wnt pathway
